# Trichobézoard gastroduodénal: à propos d’un cas

**DOI:** 10.11604/pamj.2018.30.25.12239

**Published:** 2018-05-15

**Authors:** Khalid Mazine, Pierre Barsotti, Hicham Elbouhaddouti, Ouadii Mouaqit, Elbachir Benjelloun, Khalid Ait Taleb, Abdelmalek Ousadden

**Affiliations:** 1 Université Sidi Mohamed Ben Abdellah, Service de Chirurgie Viscérale A (C3), CHU Hassan II Fes, Maroc; 2 Service de Chirurgie Generale Digestive et Endocrine Hôpital Emile Müller Mulhouse, France

**Keywords:** Trichobézoard, bézoard, trichophagie, gastroduodénal, exérèse chirurgi-cale, Trichobezoar, bezoar, trichophagy, gastroduodenal, surgical resection

## Abstract

Le trichobézoard gastroduodénal est une affection rare, dont le diagnostic est facile en présence d'un contexte évocateur. Nous rapportons le cas d'une jeune patiente âgée de 21 ans suivie pour une schizophrénie, admise pour douleur abdominale aiguë, vomissements et masse épigastrique, le scanner abdominal a permis de faire suspecter un bézoard en objectivant des lésions hétérogènes occupant tout l'estomac, ne prenant pas le contraste et semblant indépendante de la paroi gastrique. Un traitement chirurgical a été réalisé avec exérèse du trichobézoard par gastrotomie, sans complications et un transfert en service de psychiatrie.

## Abstract (en)

Gastroduodenal trichobezoar ulcer is rare. Its diagnosis is easy in the presence of an evocative context. We report the case of a 21-year old patient followed up for schizophrenia, admitted with acute abdominal pain, vomiting and epigastric mass. Abdominal CT scan suggested the diagnosis of bezoar on the basis of heterogeneous, non-enhanced lesions occupying the entire stomach and appearing to be separated from the gastric wall. Surgical removal of trichobezoar by gastrotomy was performed without complications. The patient was referred to the Department of Psychiatry.

## Introduction

Le terme bézoard désigne des corps étrangers variés retrouvés dans le tractus gastro-intestinal. La plupart se forment dans l'estomac par l'accumulation de substances non digestibles, telles certaines fibres végétales (phytobézoard), des cheveux (trichobézoard), des produits laitiers concentrés (lactobézoard), plus rarement certains médicaments (pharmacobézoard) [1]. Les trichobézoards résultent habituellement de l'accumulation de cheveux, mais, dans des rares cas, il peut s'gir de papier mâché, de laine provenant des tapis ou de vêtements [1]. Bien que rare, mais non exceptionnel, le trichobézoard concerne habituellement les enfants ou les jeunes adolescentes présentant des troubles psychiques [2]. Nous rapportons un cas de trichobézoard gastroduodénal colligé dans le service de chirurgie générale digestive et endocrine du GHR Mulhouse.

## Patient et observation

**Figure 1:**
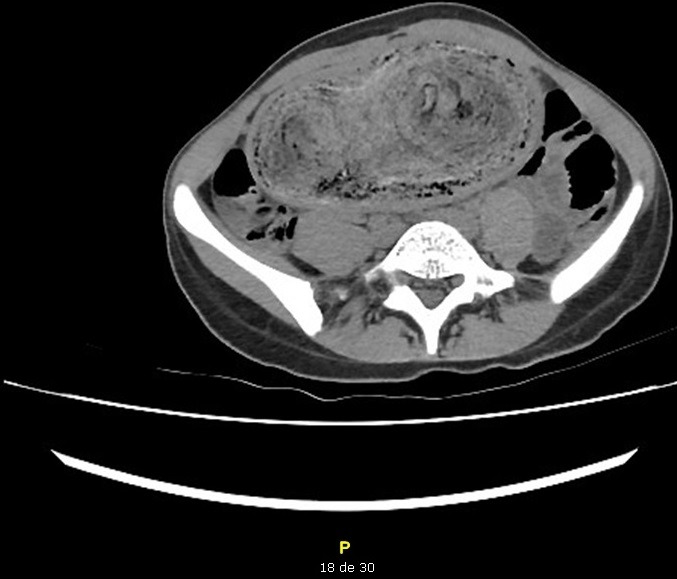
Coupe scanographie montrant le bézoard occupant l’estomac

**Figure 2:**
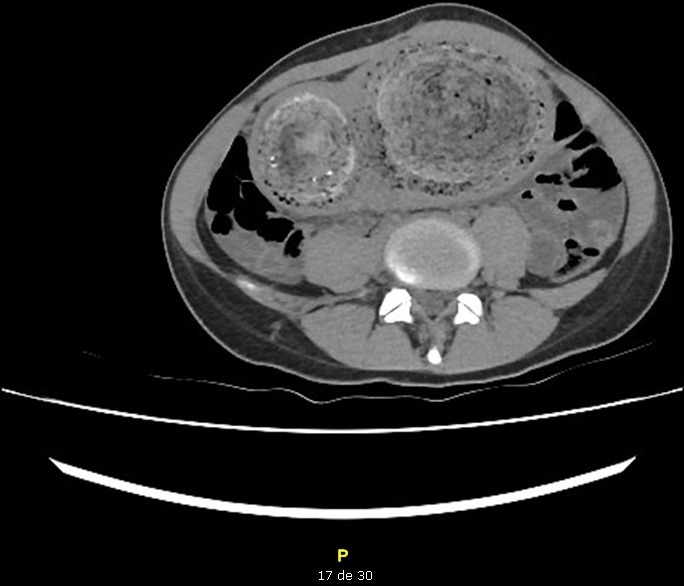
Coupe scanographie montrant le bézoard arrivant jusqu’au duodénum

**Figure 3:**
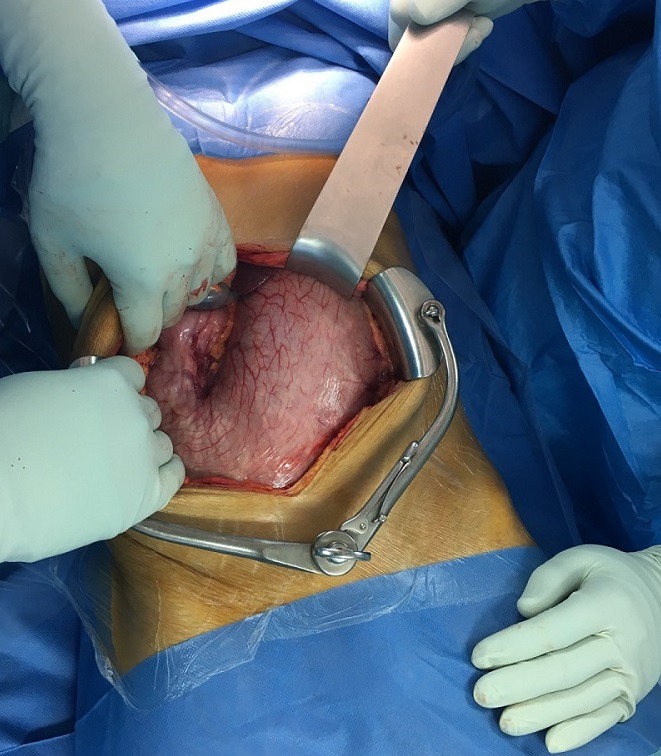
Estomac distendu inflame

**Figure 4:**
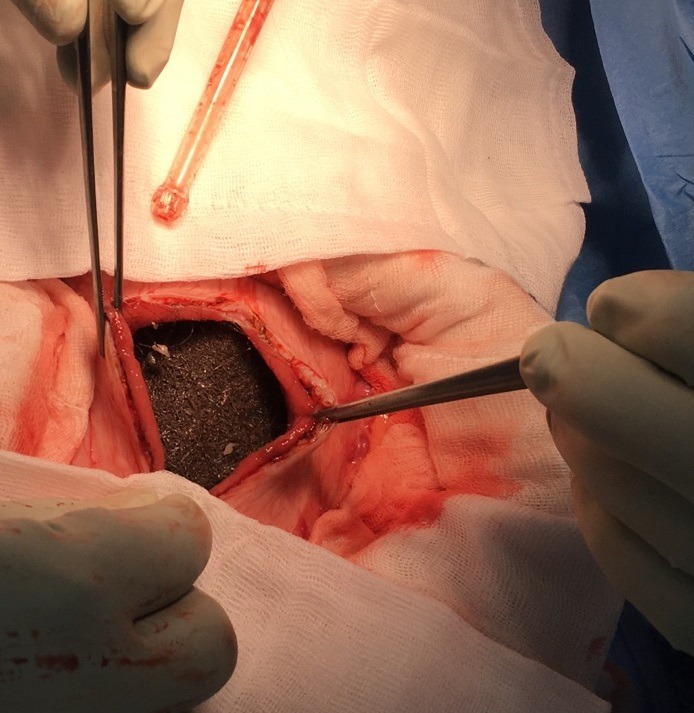
Gastrotomie

**Figure 5:**
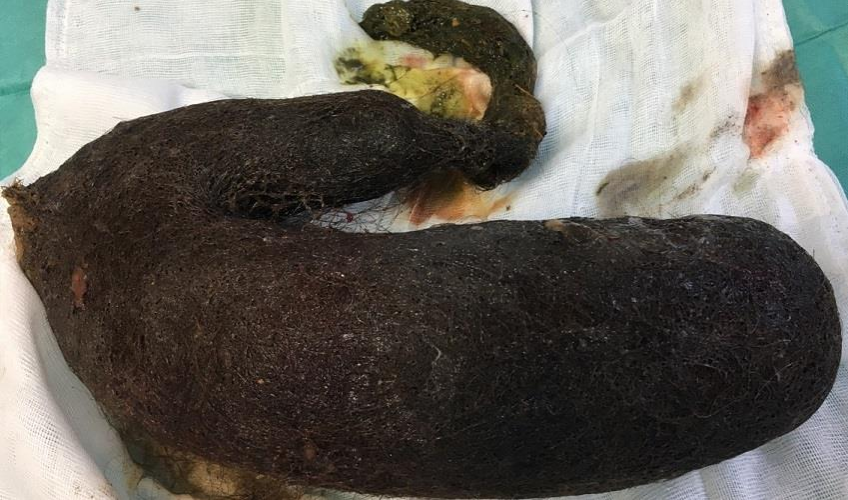
Trichobézoard gastroduodénal

Une jeune patiente âgée de 21ans, suivie pour schizophrénie depuis plusieurs années avec de nombreux séjours en service de psychiatrie. Admise aux urgences chirurgicales pour douleurs abdominales diffuses, associées à des vomissements alimentaires postprandiaux sans troubles de transit, cette symptomatologie évolue depuis 8j. L'entourage de la patiente rapporte par ailleurs une anorexie et un amaigrissement chiffré à 10kg en 3 mois. L'examen clinique trouve une patiente en assez bon état général, consciente, stable hemodynamiquement, on note par ailleurs, une plaque d'alopécie frontale et temporale avec des conjonctives décolorées et une haleine fétide. L'examen abdominal a trouvé une sensibilité abdominale et une masse épigastrique, dure, mobile et douloureuse étendue à l'hypochondre droit. Le reste de l'examen clinique était sans particularités. Le bilan biologique objectivait une hyperleucocytose à prédominance PNN, une anémie hypochrome microcitaire, le reste du bilan était normal. Une TDM abdominale (Figure 1, Figure 2) a montré une distension de l'estomac siège d'une lésion hétérogène qui occupe tout le corps gastrique, l'antre ainsi que le cadre duodénal, elle mesure 23 cm de hauteur, 13 cm de diamètre transversale et 7 cm d'épaisseur ne se rehaussant pas au produit de contraste et ne présentant et semblant totalement indépendante de la paroi gastrique. La patiente a été opérée Figure 3, Figure 4), elle a bénéficié d'une exérèse chirurgicale du trichobézoard gastroduodenal à travers une gastrotomie antérieure longitudinale (Figure 5). Les suites opératoires étaient simples avec un transfert au service de psychiatrie à j6.

## Discussion

Le trichobézoard est une affection rare, et chez l'enfant, il représente 0,15% des corps étrangers gastro-intestinaux. Le sexe féminin est le plus touché (90% des cas) et l'âge de survenue est dans 80% des cas inférieurs à 30 ans, avec un pic d'incidence entre dix et 19 ans [3], notre patiente est aussi incluse dans cette tranche d'âge. Il est important à noter que certains facteurs environnementaux et psychologiques peuvent constituer un terrain prédisposant au développement des trichobézoards. La localisation gastrique est la plus fréquente, les boucles de cheveux ainsi ingérées sont attrapées par la muqueuse gastrique à laquelle elles se fixent et forment un enchevêtrement plus ou moins complexe, une sorte de grillage au niveau duquel s'agglomèrent les aliments, réalisant une masse compacte intimement fixée à la paroi gastrique. Le trichobézoard ainsi formé peut s'étendre à l'intestin grêle arrivant parfois à la dernière anse iléale, voire au côlon transverse, réalisant ainsi le syndrome de Rapunzel [4,5]. Chez notre patiente, il s'agit d'un trichobézoard intéressant l'estomac et le duodénum. Cette affection peut rester longtemps asymptomatique, ce qui explique le retard du diagnostic qui peut aller jusqu'à plusieurs années. La symptomatologie clinique est très variée, et non spécifique [6]. Les troubles digestifs sont les plus fréquents et com-portent des douleurs abdominales essentiellement épigastrique, nausées, vomissements, diarrhée, constipation, œsophagite peptique et parfois une odeur in-supportable de l'haleine par putréfaction alimentaire, l'anorexie et l'amaigrissement peuvent être parfois l'élément clinique majeur. Il peut être révélé d'emblée par une complication aiguë; telle qu'une hémorragie digestive, une occlusion intestinale aiguë, une perforation digestive, ou une pancréatite aiguë imputée à une obstruction de l'ampoule de Vater par un prolongement du trichobézoard ou à l'œdème réactionnel, un ictère cholestatique, un ulcère gastrique ou duodénal et rarement un volvulus du gros intestin [2,4,7]. L'examen clinique, en dehors des complications, trouve grâce à une palpation soi-gneuse, une masse abdominale localisée le plus souvent au niveau de l'hypocondre gauche et/ou de l'épigastre qui ne doit pas manquer au diagnostic [8].

La découverte d'une plaque d'alopécie localisée, de caractère mécanique, est un signe d'orientation majeur et doit faire rechercher une trichophagie. Une fois le diagnostic de trichobézoard est évoqué, l'examen de choix à demander est la fibroscopie œso-gastro-duodénale, qui peut avoir un intérêt diagnostique et thérapeutique dans les formes localisées gastrique et de petite taille, elle permet à la fois de confirmer le diagnostic et d'assurer l'extraction du corps étranger, elle visualise des cheveux enchevêtrés généralement de couleur noir mais une modification de couleur peut avoir lieu due à l'effet chimique de l'acidité gastrique, aspect pathognomonique du trichobézoard [7]. Dans le trichobézoard géant, la fibroscopie est insuffisante, elle ne permet pas d'évaluer l'extension au niveau des anses jéjuno-iléales, dans ces cas, l'imagerie re-trouve tout son intérêt. Le trichobézoard apparaît en tomodensitométrie (TDM) sous forme d'une masse de volume variable, hétérogène, occupant presque la totalité de la lumière gastrique et constituée d'une multitude de cercles concentriques de densités différentes réparties en bulbes d'oignon. Deux signes pathognomoniques et constants sont la présence de bulles d'air minuscules dispersées au sein de la masse et l'absence de toute attache de celle-ci à la paroi gastrique [1,4]. L'imagerie par résonance magnétique (IRM) permet également de faire le diagnostic La masse est de signal variable en pondération T1 et T2 et ne prend pas le contraste après injection de gadolinium [8]. La prise en charge thérapeutique dépend de la taille et de la présence ou non de complications. Pour un trichobézoard gastrique de petite taille, il est généralement extirpable endos-copiquement, mais s'il est volumineux et entendu aux anses ou au stade de complications la chirurgie est le traitement de choix [7-10], comme c'était le cas chez notre patiente. La laparotomie avec extraction du trichobézoard par gastrotomie éventuellement complété d'une entérotomie est le traitement des formes étendues et des cas échéants du traitement endoscopique. Une prise en charge psychiatrique, à base de thérapie comportementale, d'éducation parentale et de traitement médical, doit souvent être instaurée chez les patients pré-sentant une trichophagie.

## Conclusion

Le trichobézoard est une pathologie rare qui survient habituellement chez des adolescents présentant des troubles psychiques. La symptomatologie clinique est très variée. Le diagnostic est suspecté devant l'association de l'alopécie, la trichomanie et de trouble digestif et sa confirmation repose sur les données de la fibroscopie œsogastro-duodénale et l'imagerie qui oriente ainsi la conduite thérapeutique. En plus de la chirurgie, la prise en charge psychologique est un temps essentiel dans le traitement et surtout la prévention des récidives.

## Conflits d’intérêts

Les auteurs ne déclarent aucun conflit d'intérêts.
